# Diagnostic accuracy of a machine learning-based radiomics approach of MR in predicting IDH mutations in glioma patients: a systematic review and meta-analysis

**DOI:** 10.3389/fonc.2024.1409760

**Published:** 2024-07-30

**Authors:** Xiaoli Chen, Junqiang Lei, Shuaiwen Wang, Jing Zhang, Lubin Gou

**Affiliations:** ^1^ Department of Radiology, The First Hospital of Lanzhou University, Lanzhou, China; ^2^ Intelligent Imaging Medical Engineering Research Center of Gansu Province, Lanzhou, China; ^3^ Accurate Image Collaborative Innovation International Science and Technology Cooperation Base of Gansu Province, Lanzhou, China; ^4^ Department of MRI, Shaanxi Provincial People’s Hospital, Xi’an, China

**Keywords:** glioma, isocitrate dehydrogenase (IDH), MRI, machine learning, deep learning, radiomics

## Abstract

**Objectives:**

To assess the diagnostic accuracy of machine learning (ML)-based radiomics for predicting isocitrate dehydrogenase (IDH) mutations in patients with glioma.

**Methods:**

A systematic search of PubMed, Web of Science, Embase, and the Cochrane Library from inception to 1 September 2023, was conducted to collect all articles investigating the diagnostic performance of ML for the prediction of IDH mutations in gliomas. Two reviewers independently screened all papers for eligibility. Methodological quality and risk of bias were assessed using the METhodological RadiomICs Score and Quality Assessment of Diagnostic Accuracy Studies-2, respectively. The pooled sensitivity, specificity, and 95% confidence intervals were calculated, and the area under the receiver operating characteristic curve (AUC) was obtained.

**Results:**

In total, 14 original articles assessing 1740 patients with gliomas were included. The AUC of ML for predicting IDH mutation was 0.90 (0.87–0.92). The pooled sensitivity, specificity, and diagnostic odds ratio were 0.83 (0.71–0.90), 0.84 (0.74–0.90), and 25 (12,50) respectively. In subgroup analyses, modeling methods, glioma grade, and the combination of magnetic resonance imaging and clinical features affected the diagnostic performance in predicting IDH mutations in gliomas.

**Conclusion:**

ML-based radiomics demonstrated excellent diagnostic performance in predicting IDH mutations in gliomas. Factors influencing the diagnosis included the modeling methods employed, glioma grade, and whether the model incorporated clinical features.

**Systematic review registration:**

https://www.crd.york.ac.uk/PROSPERO/#myprospero, PROSPERO registry (CRD 42023395444).

## Introduction

The 2016 World Health Organization (WHO) classification of central nervous system tumors incorporated molecular markers (1). The 2021 WHO classification emphasizes the role of molecular markers in both the classification and grading of gliomas (2). The primary markers for gliomas include isocitrate dehydrogenase (IDH), classified as IDH-mutant, 1p/19q-non-codeleted (IDHmut-Noncodel), and IDH wild-type (IDHwt). Patient outcomes and therapeutic options in glioma vary across subtypes (3, 4). Patients with an IDH-mutated glioma have a better prognosis than those with an IDH wild-type tumor. Recent studies have demonstrated that IDH may be a potential therapeutic target for IDH-mutant gliomas (5). Therefore, preoperative prediction of IDH mutation status is important for prognosis and therapeutic decision-making. Although histopathology is the current diagnostic gold standard, it has some limitations such as sampling errors, complications, and invasiveness. Thus, noninvasive assessment of IDH mutation status is an urgent requirement.

Radiomics can transform images into mineable data for quantitative analysis through high-throughput extraction and analysis, providing support for decision-making (6). Machine learning and deep learning combined with radiomics have excellent potential for preoperative diagnosis, staging, and therapeutic effect evaluation of gliomas (7, 8), as well as for predicting IDH mutation status. A previous systematic review (9) dealing with this subject was published, but it was not quantitative enough to evaluate the predictive performance. In addition, because radiomics research is a complicated process that includes multiple stages, it is critical to evaluate the quality of the method to ensure the reliability and reproducibility of the model before use in clinical work.

The aim of this systematic review and meta-analysis was to evaluate the accuracy of radiomics models in predicting the IDH status of gliomas and to evaluate the methodological quality and risk of bias in radiomics workflows.

## Materials and methods

This meta-analysis was performed according to the Preferred Reporting Items for Systematic Reviews and Meta-Analyses (10) guidelines and registered to the PROSPERO registry (registration number, CRD 42023395444).

### Literature search and study selection

The PubMed, EMBASE, Cochrane Library, and Web of Science databases were searched up to 1 September 2023 by two reviewers, C.X.L and Z.J. To identify the relevant articles, only English articles were considered. The following keywords were used to identify relevant studies: (“Glioma” OR “Gliomas”) AND (“Isocitrate Dehydrogenase” OR “ IDH”) AND (“MRI” OR “magnetic resonance imaging”) AND (“machine learning” OR “radiomics” OR “deep learning” OR “Artificial Intelligence”) The details of search strategies are provided in the **Supplementary Materials**.

The included articles fulfilled all the following criteria: 1) patients with pathologically confirmed glioma; 2) histopathological examination with the IDH mutation as a reference standard; 3) sufficient data for the reconstruction of 2×2 tables in terms of the diagnostic performance of MR-based radiomics in predicting the IDH of glioma; and 4) original research articles. The exclusion criteria were as follows: 1) each study had at least 10 patients; 2) reviews, case reports, letters, and editorials; 3) studies not focusing on the diagnostic performance of MR-based radiomics in predicting IDH mutations; and 4) insufficient data for the reconstruction of 2×2 table studies with overlapping cohorts. Two authors, C. X.L and Z.J, independently evaluated the eligibility of the included articles, and any disagreements were resolved via discussion with a third author (W.S.W, with 10 years of experience in neuroimaging).

### Quality assessment and data extraction

The included articles’ methodological quality and the risk of bias at the study level were assessed using the Quality Assessment Tool for Diagnostic Accuracy Studies (QUADAS)-2 (11) and METhodological RadiomICs Score (12), respectively. The QUADAS-2 tool included four parts: (a) patient selection, (b) index test, (c) reference standard, and (d) flow and timing. The METhodological RadiomICs Score (METRICS tool included 30 items within 9 categories for evaluating the quality of the radiomics workflow. Two reviewers, C.X.L and G.L.B, assessed the quality of the articles separately and resolved any disagreements through discussion with a third author (W.S.W).

The following data were extracted from the included articles: 1) study characteristics (authors, year of publication, country of origin, study design (prospective vs. retrospective)); 2) patient and clinical characteristics (number of patients, age, WHO grade, reference standard); 3) technical characteristics of magnetic resonance imaging (MRI) (magnetic field strength (T), scanner, scan sequence) and machine learning details (classifier, method of segmentation, VOI or ROI, and external or internal validation).

### Statistical analysis

This meta-analysis was performed using Stata 16 Review Manager 5.3 software and Meta-disc 1.4. Pooled sensitivity, specificity, diagnostic odds ratio (DOR), positive likelihood ratio (PLR), and negative likelihood ratio (NLR) with 95% confidence intervals (CIs) were calculated using bivariate random effects, and a summary receiver operating characteristic (SROC) curve and area under the curve (AUC) were generated to illustrate the diagnostic performance.

The heterogeneity of the included studies was calculated using the Q-test (p value ≤ 0.05) and I^2^ statistic (>50%) (13). A Spearman coefficient >0.6 indicated the threshold effect (14). Subgroup analysis was performed to further investigate the potential cause of heterogeneity, and the following four covariates were included: 1) machine learning (ML) vs. deep learning (DL), 2) only radiomics vs. combination of radiomics and clinical information, 3) low-grade glioma (LGG) vs. high-grade glioma (HGG), and 4) external validation vs. internal validation.

## Results

### Characteristics of included studies

**Figure 1:**
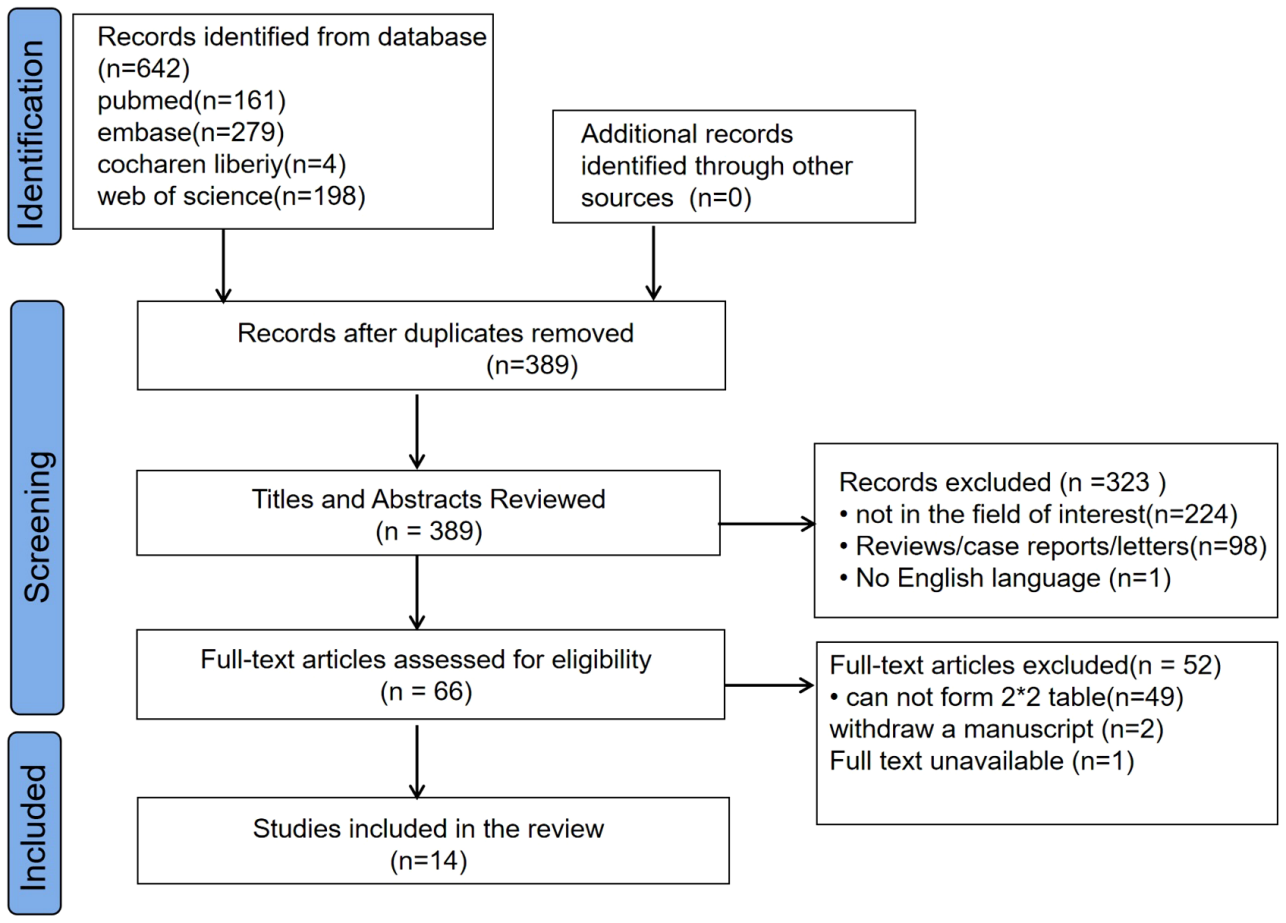
Flow chart of study selection.

The flowchart of the literature search and selection process is displayed in **Figure 1**, which yielded 161 studies from PubMed, 279 from Embase, four from the Cochrane Library, and 198 from the Web of Science. After removing 253 duplicate articles, the remaining 389 articles were screened for their title and abstract. The full text of 66 eligible articles was reviewed, and 14 articles (15–28) were included in this meta-analysis.

The characteristics of the included studies are shown in **Tables 1** and **2**. One study was prospective, and the remaining studies were retrospective. Eight (15–17, 20–22, 26, 27) of the 14 studies used 3-TMRI, four (18, 23, 25, 28) used 1.5-T or 3-TMR, and two (19, 24) used 1.5T. Among these, 14 included studies, 12 (16–23, 25–28) used radiomics combined with ML, while two (15, 24) used DL assessment. The most commonly used ML classifiers were SVM and RFC. In addition, five (15, 16, 23, 24, 26) of the 14 studies employed external validation, three (17, 25, 28) had no validation set, and the remaining six studies (18–21, 25, 27) used internal validation. In terms of glioma grade, three studies (15, 16, 18) were low-grade gliomas, and three studies (19, 20, 25) were high-grade gliomas, whereas the remaining studies included both low- and high-grade gliomas. For the ML analysis, eleven (15–23, 25, 26, 28) studies included only radiomics information, and three (22, 24, 27) used radiomics and clinical information.

**Table 1:** Basic characteristics and details of the 14 included studies (1).

Study	Country	Study design	No. of patients	Mean age	MRI field intensity	Vendor Scanner	Sequences
Li 2017 (15)	China	Retrospective	151	40.7 ± 10.8	3T	Siemens Trio	T1CE,T2 flair
Yu 2016 (16)	China	Retrospective	110	40.3 ± 11.3 (years)	3T	Siemens Trio	T2 flair
Bisdas 2018 (17)	UK	Prospective	37	63.2 ± 7.6	3T	Siemens Skyra	T1,T1CE T2 flair,DKI
Zhang 2018 (18)	China	Retrospective	103	43.5 ± 12.9	1.5T(37),3T(66)	NA	T1,T2, T1CE T2 flair
Deniz Alis 2019 (19)	Turkey	Retrospective	142	40.87 ± 12.25	1.5T	Siemens Avanto	T1CE,T2 flair,DWI
Niu 2020 (20)	China	Retrospective	182	44 ± 11	3T	GE SIGNA	T1CE
Cao 2020 (21)	China	Retrospective	102	44.6 ± 14.9	3T	GE SIGNA	T1,T2, T1CE T2 flair,DWI
Huang 2021 (22)	China	Retrospective	59	46 ± 15	3T	Siemens MAGNETOM	T1,T2, T1CE T2 flair
Manikis 2021 (23)	Greece	Retrospective	160	58.4 ± 15.9	1.5T,3T	GE,Siemens,Philip	T1,T2, T1CE T2 flair,DCE-MR
Hrapşa 2022 (24)	Romania	Retrospective	21	48.6 ± 15.6	1.5T	GE,Siemens,TOSHIBA	T2, T1CE T2 flair
Kandalgaonkar 2022 (25)	United States	Retrospective	100	52	1.5T,3T	GE,Philip	T2, T1CE
Wang 2022 (26)	China	Retrospective	140	40	3T	Siemens	T1,T2,T1CE,T2 FLAIR, DWI
Wang, J 2022 (27)	China	Retrospective	100	48 ± 13	3T	GE	T1,T2, T1CE T2 flair,DWI,DCE-MR
CaroleH 2020 (28)	UK	Retrospective	333	NA	1.5T,3T	GE, Siemens	DSC-MR

NA, not available.

**Table 2:** Basic characteristics and details of the 14 included studies (2).

Study	WHO Grade	reference standard	Machine learning classifier	Validation	Segmentation	Region/volume of interest
Li 2017 (15)	grade 2	Sanger sequencing	CNN	External validation	Automatic	VOI
Yu 2016 (16)	grade 2	Sanger sequencing	SVM and AdaBoost	External validation	Automatic	VOI
Bisdas 2018 (17)	grade 2,3	Sanger sequencing	SVM	No validation	Manual	VOI
Zhang 2018 (18)	grade 2,3	NA	SVM	Internal validation	Manual	VOI
Deniz Alis 2019 (19)	grade 3,4	Histopathological	RFC	Internal validation	Manual	VOI
Niu 2020 (20)	grade 3,4	Immunohistochemistry	biclassification mode	Internal validation	Manual	ROI
Cao 2020 (21)	grade 2,3	Histopathological	RFC	Internal validation	Manual	VOI
Huang 2021 (22)	grade 2,3	DNAsingle-step assay	Multivariate logistic regression	No validation	Manual	VOI
Manikis 2021 (23)	grade 2,3,4	histologically	SVM,RF,KNN,LR,AdaBoost	External validation	Automatic	VOI
Hrapşa 2022 (24)	grade 2,3,4	MLPA-Multiplex PCR) or immunohistochemistry	CNN	External validation	Automatic	VOI
Kandalgaonkar 2022 (25)	grade 4	Immunohistochemistry, Sanger sequencing	SVM,10-fold cross-validation	No validation	Manual	ROI
Wang 2022 (26)	grade 2,3,4	Histologically	ANN	External validation	Segmentation and manual	ROI
Wang, J 2022 (27)	NA	IDH1 R132H mutation-specific antibody	Liner SVM	Internal validation	Automatic	ROI
CaroleH 2020 (28)	grade 2,3,4	Histopathology	Random-forest algorithm	No validation	Manual	VOI

NA, not available.

### Quality assessment

**Figure 2:**
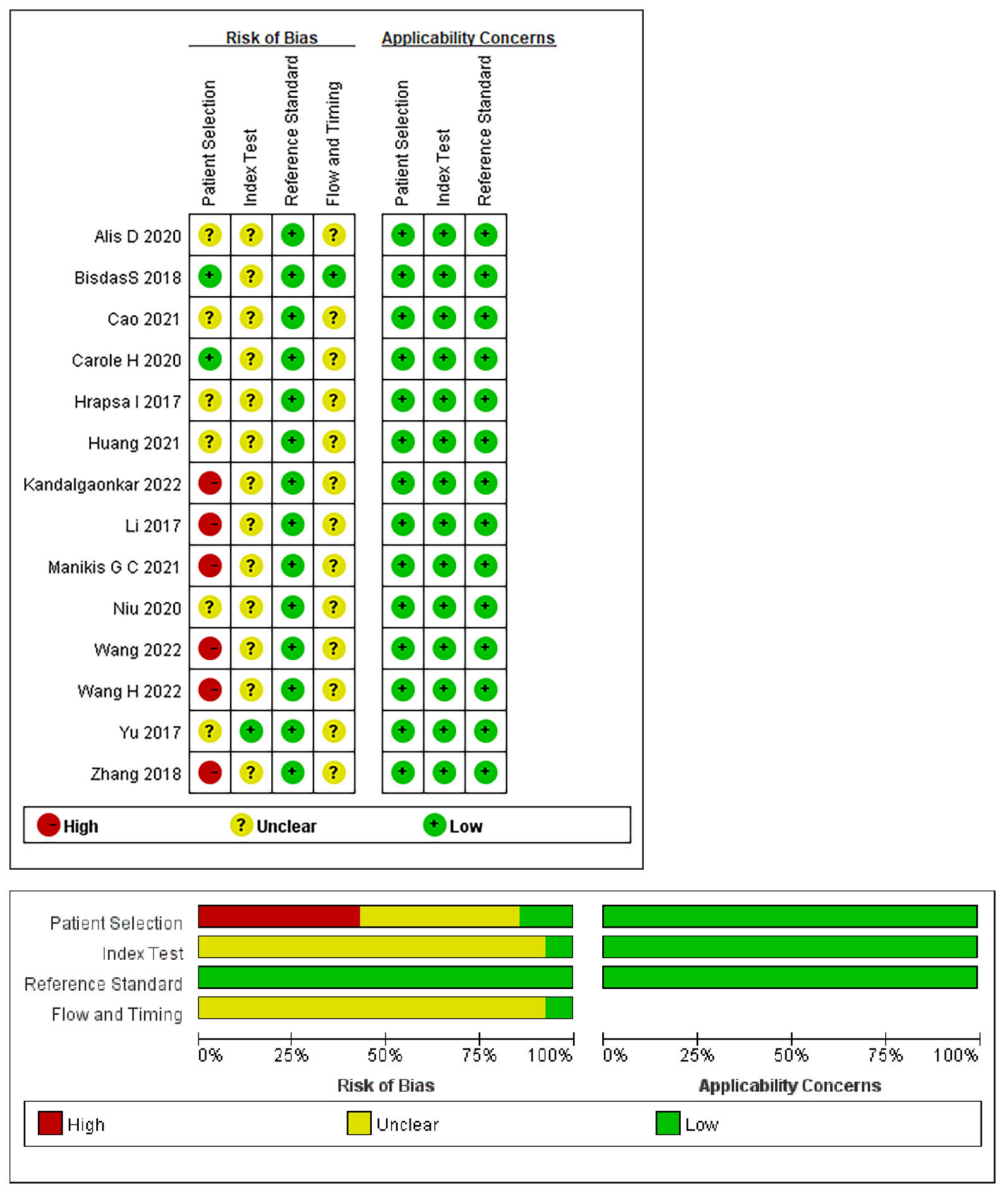
Summary of the risk of bias and applicability assessments: the authors’ judgements for each domain of each included study were reviewed. The proportion of included studies that indicated low, unclear, or high risk and applicability concerns are shown in green, yellow, and red, respectively.

The risk of bias and applicability assessment of the included studies, performed using the QUADAS-2 tool, are shown in **Figure 2**. In terms of the patient selection, two (17, 28) studies were deemed to have a low risk of bias, six (15, 18, 23, 25–27) exhibited a high risk of bias owing to unclear information regarding the time range and consecution of patients, and six (16, 19–22, 24) were considered to have an unclear risk because of uncertainties in the consecution of patients. Regarding the index test, 13 studies had an unclear risk of bias owing to ambiguity regarding the use of a threshold. All the studies indicated a low risk of bias in the reference standards. Regarding flow and timing, 13 studies had an unclear risk of bias because there was no mention of the time interval between imaging and molecular testing.

The mean METRICS score of the included studies was 60.3% (range, 50%-75%), the quality of six (15–17, 22, 25, 28) studies was moderate (40≤score<60%),and eight studies (17–21, 23, 24, 26) were good (60≤score<80%). The highest score of 75% was obtained in one study (26) and the lowest score of 50% was observed in two studies (22, 25), primarily attributed to a lack of a validation cohort. The item of “Model availability” was assigned zero points as none of the included studies addressed it. Only one study (24) publicly shared the code. A detailed description of the METRICS scores is provided in **Table 3**.

**Table 3:** METRICS of the included studies.

Categories	No.	Wang, J.2022 (27)	Wang, H.2022 (26)	Kandalgaonkar, P.2022 (25)	Yu, J.2017 (16)	Hrapsa,I 2022 (24)	Niu L 2020 (20)	Cao, M. 2021 (21)	Bisdas, S.2018 (17)	Li, Z 2017 (15)	Zhang Xi, 2018 (18)	Alis, D.2020 (19)	Huang, W. Y.2021 (22)	Manikis, G. C.2021 (23)	Carole H. Sudre.2020 (28)
Study Design	#1	0.0368	0.0368	0.0368	0.0368	0.0368	0.0368	0.0368	0	0.0368	0.0368	0.0368	0.0368	0.0368	0.0368
	#2	0.0735	0.0735	0.0735	0.0735	0.0735	0.0735	0.0735	0.0735	0.0735	0.0735	0.0735	0.0735	0.0735	0.0735
	#3	0.0919	0.0919	0.0919	0.0919	0.0919	0.0919	0.0919	0.0919	0.0919	0.0919	0.0919	0.0919	0.0919	0.0919
Imaging Data	#4	0	0.0438	0	0	0	0	0	0	0	0	0	0	0.0438	0.0438
	#5	0.0292	0.0292	0.0292	0.0292	0.0292	0.0292	0.0292	0.0292	0.0292	0.0292	0.0292	0.0292	0.0292	0.0292
	#6	0.0438	0.0438	0.0438	0.0438	0.0438	0.0438	0.0438	0.0438	0.0438	0.0438	0.0438	0.0438	0.0438	0.0438
	#7	0.0292	0.0292	0.0292	0.0292	0.0292	0.0292	0.0292	0.0292	0.0292	0.0292	0.0292	0.0292	0.0292	0.0292
Segmentation	#8	0.0337	0.0337	0.0337	0.0337	0	0.0337	0.0337	0.0337	0.0337	0.0337	0.0337	0.0337	0.0337	0.0337
	#9	0	0	0	0.0225	0.0225	0	0	0	0.0225	0	0	0	0.0225	0
	#10	0	0.0112	0	0.0112	0.0112	0	0	0	0.0112	0	0.0112	0	0	0.0112
Image Processing and Feature Extraction	#11	0	0.0622	0	0	0.0622	0	0	0.0622	0	0	0.0622	0	0.0622	0.0622
	#12	0.0311	0.0311	0.0311	0	0.0311	0.0311	0.0311	0.0311	0	0.0311	0.0311	0.0311	0.0311	0.0311
	#13	0.0415	0.0415	0.0415	0.0415	0.0415	0.0415	0.0415	0.0415	0.0415	0.0415	0.0415	0.0415	0.0415	0.0415
Feature Processing	#14	0	0	0.02	0	0	0	0	0	0	0	0	0	0	0
	#15	0.02	0.02	0.02	0.02	0.02	0.02	0.02	0.02	0.02	0.02	0.02	0.02	0.02	0.02
	#16	0.03	0	0	0	0	0.03	0.03	0	0	0.03	0	0	0	0
	#17	0	0	0	0	0	0	0	0	0	0	0	0	0	0
Preparation for Modeling	#18	0.0599	0.0599	0	0	0	0.0599	0.0599	0	0	0.0599	0.0599	0	0	0
	#19	0	0	0	0	0	0	0	0	0	0	0	0	0	0
Metrics and Comparison	#20	0.0352	0.0352	0.0352	0.0352	0.0352	0.0352	0.0352	0.0352	0.0352	0.0352	0.0352	0.0352	0.0352	0.0352
	#21	0.0234	0.0234	0	0	0.0234	0.0234	0	0	0	0.0234	0.0234	0.0234	0	0
	#22	0.0176	0	0	0	0	0	0	0	0	0	0	0	0	0
	#23	0	0	0.0117	0.0117	0	0.0117	0	0	0	0	0	0.0117	0	0
	#24	0	0	0	0	0	0	0.0293	0	0	0	0	0	0	0
	#25	0.0176	0	0	0	0	0	0	0	0	0	0	0	0	0
Testing	#26	0.0375	0	0	0	0	0.0375	0.0375	0.0375	0	0.0375	0.0375	0	0	0
	#27	0	0.0749	0	0.0749	0.0749	0	0	0	0.0749	0	0	0	0.0749	0
Open Science	#28	0	0.0075	0.0075	0	0	0	0	0	0	0	0	0	0	0
	#29	0	0	0	0	0.0075	0	0	0	0	0	0	0	0	0
	#30	0	0	0	0	0	0	0	0	0	0	0	0	0	0
	total	65%	75%	50%	56%	63%	63%	62%	53%	54%	62%	66%	50%	67%	58%

#1 Adherence to radiomics and/or machine learning-specific checklists or guidelines; #2 Eligibility criteria that describe a representative study population ; #3 High-quality reference standard with a clear definition ; #4 Multi-center ; #5 Clinical translatability of the imaging data source for radiomics analysis ; #6 Imaging protocol with acquisition parameters ; #7 The interval between imaging used and reference standard ; #8 Transparent description of segmentation methodology ; #9 Formal evaluation of fully automated segmentation^2^; #10 Test set segmentation masks produced by a single reader or automated tool ; #11 Appropriate use of image preprocessing techniques with transparent description ; #12 Use of standardized feature extraction software^3^; #13 Transparent reporting of feature extraction parameters, otherwise providing a default configuration statement ; #14 Removal of non-robust features^4;^ #15 Removal of redundant features^4^; #16 Appropriateness of dimensionality compared to data size^4;^ #17 Robustness assessment of end-to-end deep learning pipelines^5;^ #18 Proper data partitioning process ; #19 Handling of confounding factors ; #20 Use of appropriate performance evaluation metrics for task ; #21 Consideration of uncertainty ; #22 Calibration assessment ; #23 Use of uni-parametric imaging or proof of its inferiority ; #24 Comparison with a non-radiomic approach or proof of added clinical value ; #25 Comparison with simple or classical statistical models ; #26 Internal testing ; #27 External testing ; #28 Data availability ; #29 Code availability ; #30 Model availability.

### Data analysis

**Figure 3:**
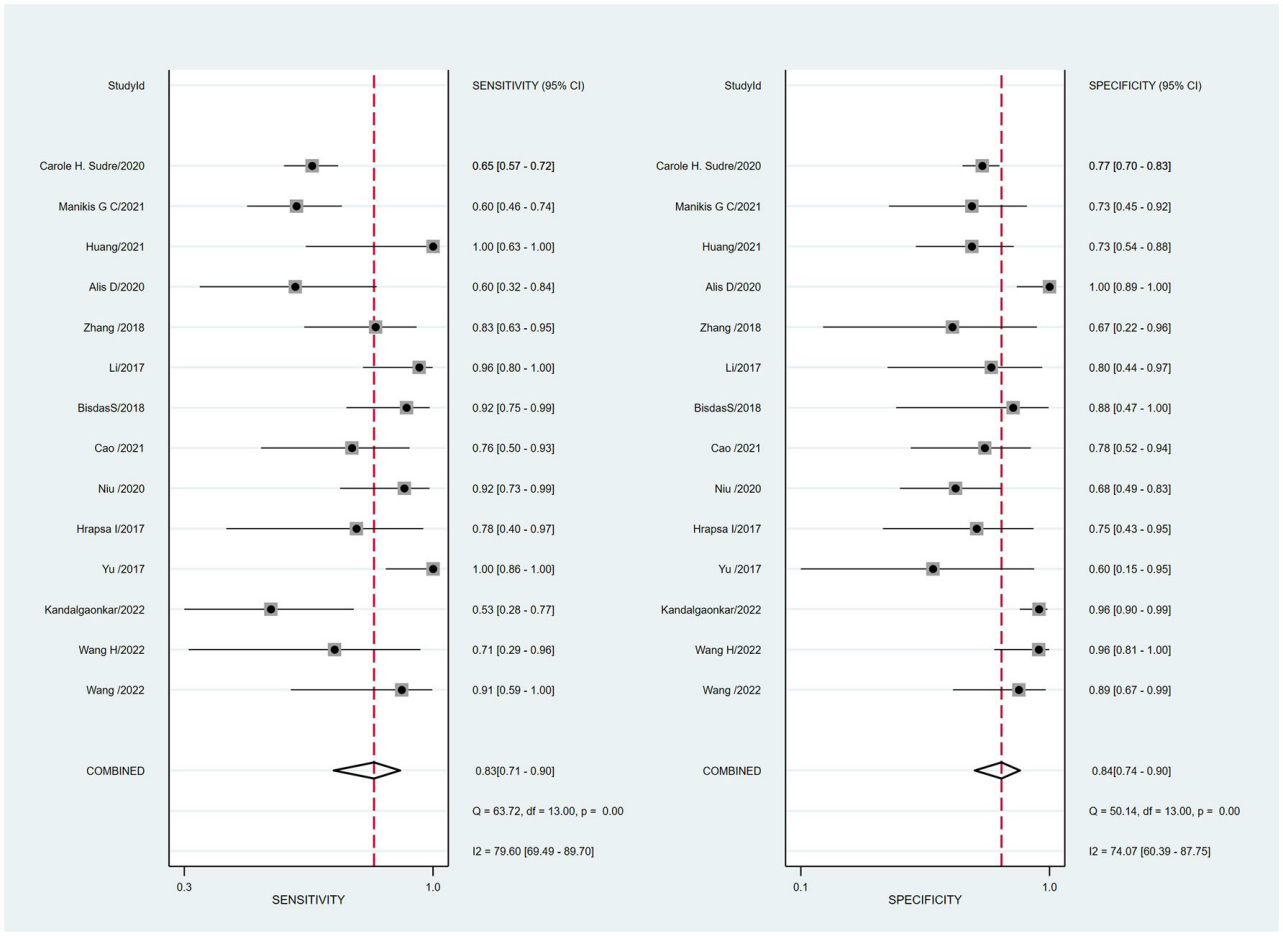
Coupled forest plots of the pooled sensitivity and specificity for the diagnostic performance of machine learning-based radiomics for the prediction of IDH mutation glioma.

**Figure 4:**
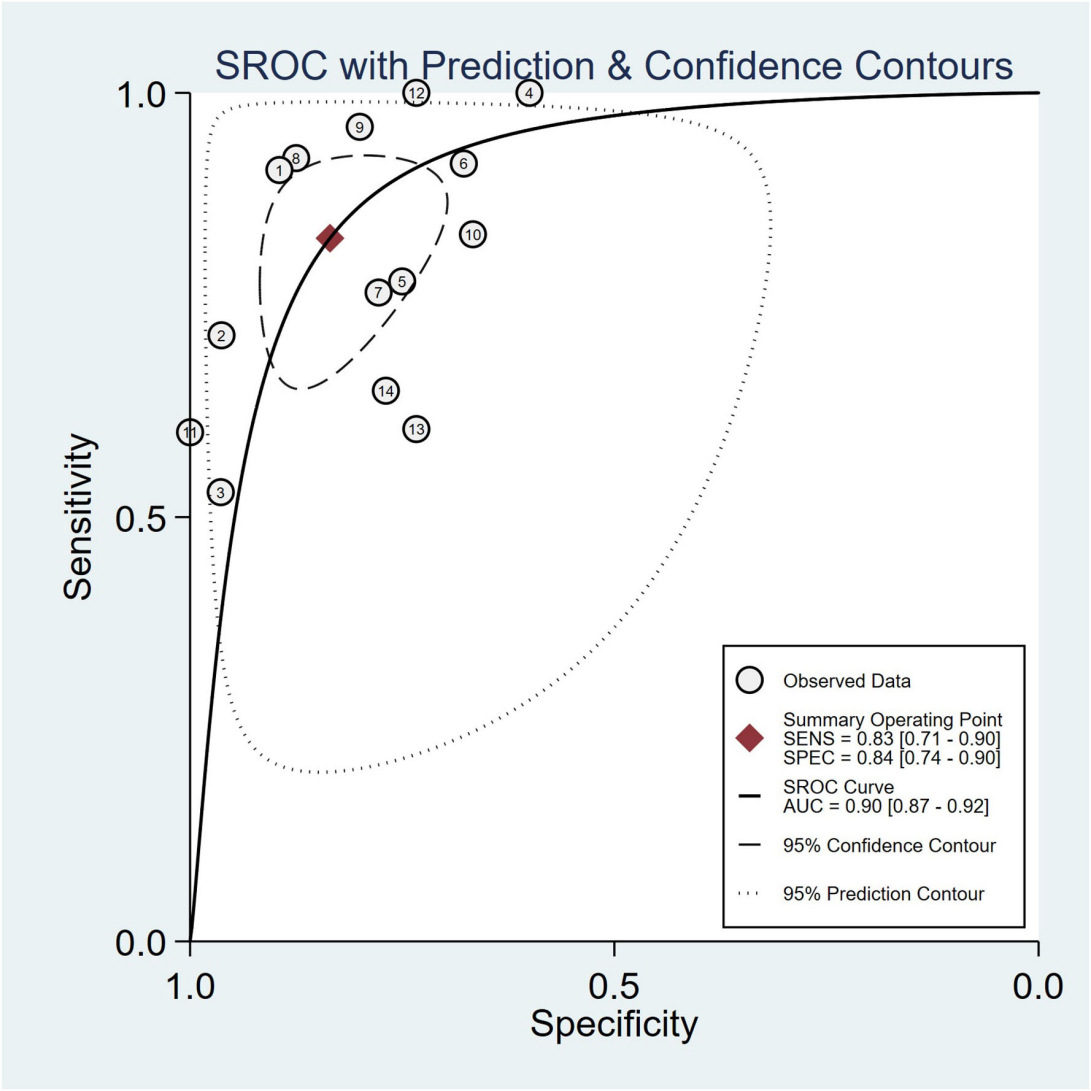
Hierarchical summary receiver operating characteristic (SROC) curve of the diagnostic performance of machine learning-based radiomics for the prediction of IDH mutation glioma.

Summaries of the ML models predicting IDH mutations in patients with glioma were analyzed using the random-effects method because of significant statistical heterogeneity (I^2^ = 92%). For all 14 studies, the pooled sensitivity, specificity, PLR, NLR, and DOR were 0.83 (0.71,0.90), 0.84 (0.74, 0.90), 5.0 (3.2, 7.8), 0.21 (0.12, 0.35), and 25 (12,50), respectively. The overall pooled AUC was 0.90 (0.87, 0.92), indicating a high diagnostic performance. Forest plots for sensitivity and specificity are illustrated in **Figure 3**, and the SROC curve is presented in **Figure 4**.

Cochran’s Q test showed significant heterogeneity (Q=25.320, p=0.00) across the studies, with a Higgins’s I^2^ statistic of 79% for sensitivity and 74.1% for specificity. The Spearman correlation coefficient between the sensitivity and false-positive rate was 0.525 (p=0.054), which indicated no threshold effect among the included studies.

### Subgroup analysis

Subgroup analysis was performed by comparing studies with different variables. **Supplementary Table 4** shows the results of the subgroup analysis. Studies using DL had a higher specificity and a lower sensitivity (0.91 [0.76, 0.98], 0.77 [0.55, 0.92]) than those using ML (0.78 [0.72, 0.83], 0.86 [0.82, 0.90]). Compared with the studies that only used radiomics features, studies combining the use of radiomics and clinical information showed higher sensitivity and lower specificity (0.89 [0.72, 0.98] vs 0.73 [0.68, 0.78], 0.79 [0.66, 0.88] vs. 0.83 [0.79, 0.86]). In addition, the sensitivity of diagnosing LGG was higher (0.93 [0.85, 0.98]) than that of diagnosing HGG (0.71 [0.58, 0.83]), but the specificity of diagnosing LGG was lower than that of diagnosing HGG (0.71 [0.48, 0.89] vs. 0.91 [0.85, 0.95]). Studies that performed external validation showed lower sensitivity and specificity than those that used internal validation (0.78 [0.70, 0.85] vs. 0.81 [0.74, 0.87]; 0.83 [0.72,0.91] vs. 0.87[0.82,0.91], respectively).

## Discussion

This systematic review and meta-analysis evaluated the diagnostic performance of radiomics in predicting IDH mutations. The pooled sensitivity, specificity, and AUC were 83% (95% CI, 0.71–0.90), 84% (95% CI, 0.74–0.90), and 0.90 (95% CI, 0.87–0.92), respectively. This indicates that radiomics combined with ML and DL could be an effective and accurate diagnostic tool for predicting IDH mutations in gliomas.

Obviously, heterogeneity was noted in the specificity (I²=79.6%) and sensitivity (I²=74.1%), Thus, we performed subgroup analysis to explore the source of the heterogeneity which included the modeling methods (ML vs. DL), glioma grade, whether the model incorporated clinical features, and validation methods (external and internal validation).The results of the present meta-analysis showed that studies using ML had a better diagnostic performance than those using DL. This could be attributed to the small sample sizes of the included studies. DL is capable of training multi-layer deep neural networks, which show significant potential in handling very large datasets with thousands or even millions of instances; however, in scenarios where the size of the dataset is small, DL tends to exhibit lower performance compared to ML. Similar findings have been previously reported for ML in other studies (29, 30). However, only two studies included in our study used DL; thus, future work should incorporate a greater number of studies with sufficient datasets to explore its true diagnostic capabilities. A previous study (31) demonstrated that the combined model of magnetic resonance (MR) and clinical features with ML exhibits better diagnostic performance than that using only MR features. Clinical features such as age, sex, and exposure to ionizing radiation were closely related to the pathological process of glioma (32, 33). For example, age is a risk factor for the development of high-grade glioma; young patients are more likely to suffer from IDH1-mutant glioma, and their postoperative survival and clinical prognosis may be more optimistic (20). Our findings are consistent with the previous study; therefore, we recommend the combined use of MR and clinical features with ML in future radiomics studies to verify their true diagnostic capabilities in predicting IDH mutation status in gliomas. The diagnostic performance in predicting the IDH mutation of HGG was better than that of LGG in the present study, which is consistent with that of a previous meta-analysis (31); however, it is essential to note that more studies are required to validate this conclusion, given the limited number of included studies. Additionally, we found that studies using external verification models had a diagnostic performance similar to that of studies using internal verification models, demonstrating the stability of the model. Internal validation tends to overestimate the diagnostic value owing to the model’s lack of generalizability (34); thus, external validation prediction models are required to reliably estimate the diagnostic capabilities of other datasets.

METRICS is a new quality assessment tool which includes 30 items within 9 categories to evaluate the key steps in the radiomics research workflow. It was developed by a large group of international experts in the field recently and is easy to use, specifically aimed at improving the methodological quality of radiomics research. The METRICS score of the included studies ranged from 50% to 75% and the mean score was 60.3%. The quality of 6 studies was moderate (40≤score<60%) and 8 studies were good (60≤score<80%). For the items with the highest weights, such as high-quality reference standards with a clear definition and eligibility criteria that describe a representative study population, all the included studies received a full score. Only one study (24) publicly shared the code and two studies (25, 26) publicly shared the data, however, these two items which belong to the “open science” category had the lowest weight. Although METRICS is a valuable tool for evaluating the quality of radiomics research, it is not without limitations. Further revision of METRICS may enhance its comprehensiveness in assessing the quality of radiomics studies.

QUADAS-2 quality assessment revealed other issues in the included studies that can be avoided in future investigations. For example, the majority of the studies did not mention the consecution of patients and the time interval between imaging and molecular testing, which led to a high or unclear bias risk. In 13 studies, it remained unclear whether thresholds were pre-specified or not, potentially resulting in an overestimation of the diagnostic performance of the models.

This study had several limitations. First, most of the included studies had a retrospective design, and only one had a prospective design; thus, selection bias was inevitable. Therefore, prospective multicenter studies with larger scales are required to validate our findings. Second, the sample size of the included studies was not large enough for training and validation, which limited the statistical power of the study and may affect the generalizability of the results. Third, significant heterogeneity was observed, which is observed in other meta-analyses of diagnostic accuracy using ML based on radiomics. Finally, the mean METRICS score of the included studies was 60.3%, indicating moderate overall quality. Therefore, further high-quality radiomics studies are required to verify our results. Despite these limitations, our review provided new insights into the accuracy of ML-based radiomics models for predicting IDH mutations in gliomas.

In conclusion, ML-based radiomics demonstrated excellent diagnostic performance for predicting IDH mutations in gliomas. Nevertheless, owing to the limitations in the quality and quantity of the included studies, caution should be exercised when applying the results, and more standardized and prospective studies are warranted to improve the application and reliability of radiomics.

## Data availability statement

The original contributions presented in the study are included in the article/**Supplementary Material**. Further inquiries can be directed to the corresponding author.

## Author contributions

XC: Conceptualization, Formal analysis, Investigation, Methodology, Writing – original draft. JL: Conceptualization, Methodology, Project administration, Supervision, Writing – review & editing. SW: Investigation, Methodology, Writing – original draft. JZ: Data curation, Methodology, Software, Writing – original draft. LG: Methodology, Software, Validation, Writing – original draft.

## References

[1] Louis DN Perry A Reifenberger G von Deimling A Figarella-Branger D Cavenee WK . The 2016 world health organization classification of tumors of the central nervous system: a summary. Acta Neuropathol. (2016) 131:803–20. doi: 10.1007/s00401-016-1545-1 27157931

[2] Kurokawa R Kurokawa M Baba A Ota Y Pinarbasi E Camelo-Piragua S . Major changes in 2021 world health organization classification of central nervous system tumors. Radiographics. (2022) 42:1474–93. doi: 10.1148/rg.210236 35802502

[3] Cancer Genome Atlas Research Network Brat DJ Verhaak RG . Comprehensive, integrative genomic analysis of diffuse lower-grade gliomas. N Engl J Med. (2015) 372:2481–98. doi: 10.1056/NEJMoa1402121 PMC4530011 26061751

[4] Kawaguchi T Sonoda Y Shibahara I Saito I Kanamori I Kumabe I . Impact of gross total resection in patients with WHO grade III glioma harboring the IDH 1/2 mutation without the 1p/19q co-deletion. J Neurooncol. (2016) 129:505–14. doi: 10.1007/s11060-016-2201-2 27401154

[5] Pellegatta S Valletta L Corbetta C Patanè M Zucca I Sirtori FR . Effective immunotargeting of the IDH1 mutation R132H in a murine model of intracranial glioma. Acta Neuropathol Commun. (2015) 3:4. doi: 10.1186/s40478-014-0180-0 25849072 PMC4359524

[6] Gillies RJ Kinahan PE Hricak H . Radiomics: Images are more than pictures, they are data. Radiology. (2016) 278:563–77. doi: 10.1148/radiol.2015151169 PMC4734157 26579733

[7] Buda M AlBadawy EA Saha A . Deep radiogenomics of lower-grade gliomas: convolutional neural networks predict tumor genomic subtypes using MR images. Radiol Artif Intell. (2020) 2:e180050. doi: 10.1148/ryai.2019180050 33937809 PMC8017403

[8] Matsui Y Maruyama T Nitta M Saito T Tsuzuki S Tamura M . Prediction of lower-grade glioma molecular subtypes using deep learning. J Neurooncol. (2020) 146:321–7. doi: 10.1007/s11060-019-03376-9 31865510

[9] Bhandari AP Liong R Koppen J Murthy SV Lasocki A . Noninvasive determination of IDH and 1p19q status of lower-grade gliomas using MRI radiomics: A systematic review. AJNR Am J Neuroradiol. (2021) 42:94–101. doi: 10.3174/ajnr.A6875 33243896 PMC7814803

[10] McInnes MDF Moher D Thombs BD McGrath TA Bossuyt PM Clifford T . Preferred reporting items for a systematic review and meta-analysis of diagnostic test accuracy studies: The prisma-dta statement. Jama. (2018) 319:388–96. doi: 10.1001/jama.2017.19163 29362800

[11] Whiting PF Rutjes AWS Westwood ME Mallett S Deeks JJ Reitsma JB . Quadas-2: A revised tool for the quality assessment of diagnostic accuracy studies. Ann Intern Med. (2011) 155:529–36. doi: 10.3760/cma.j.issn.0254-6450.2018.04.028 22007046

[12] METhodological RadiomICs Score (METRICS): a quality scoring tool for radiomics research endorsed by EuSoMII. Insights into Imaging. (2024) 15. doi: 10.1186/s13244-023-01572-w PMC10792137 38228979

[13] Higgins JPT Thomas J Chandler J . Cochrane Handbook for Systematic Reviews of Interventions, Version 6.2 (2021). Available online at: https://training.cochrane.org/handbook/current/chapter-10#section-10-10-2 (Accessed 17 October 2021).

[14] Deville WL Buntinx F Bouter LM . Conducting systematic reviews of diagnostic studies: didactic guidelines. BMC Med Res Methodol. (2002) 2:9. doi: 10.1186/1471-2288-2-9 12097142 PMC117243

[15] Li Z Wang Y Yu J Guo Y Cao W . Deep Learning based Radiomics (DLR) and its usage in noninvasive IDH1 prediction for low grade glioma. Sci Rep. (2017) 7:5467. doi: 10.1038/s41598-017-05848-2 28710497 PMC5511238

[16] Yu J Shi Z Lian Y Li Z Liu T Gao Y . Noninvasive IDH1 mutation estimation based on a quantitative radiomics approach for grade II glioma. Eur Radiol. (2017) 27:3509–22. doi: 10.1007/s00330-016-4653-3 28004160

[17] Bisdas S Shen H Thust S Katsaros V Stranjalis G Boskos C . Texture analysis- and support vector machine-assisted diffusional kurtosis imaging may allow in *vivo* gliomas grading and IDH-mutation status prediction: a preliminary study. Sci Rep. (2018) 8:6108. doi: 10.1038/s41598-018-24438-4 29666413 PMC5904150

[18] Zhang X Tian Q Wang L Liu Y Li B Liang Z . Radiomics strategy for molecular subtype stratification of lower-grade glioma: detecting IDH and TP53 mutations based on multimodal MRI. J Magn Reson Imag. (2018) 48:916–26. doi: 10.1002/jmri.25960 29394005

[19] Alis D Bagcilar O Senli YD Yergin M Isler C Kocer N . Machine learning-based quantitative texture analysis of conventional MRI combined with ADC maps for assessment of IDH1 mutation in high-grade gliomas. Jpn J Radiol. (2020) 38:135–43. doi: 10.1007/s11604-019-00902-7 31741126

[20] Niu L Feng WH Duan CF Liu YC Liu JH Liu XJ . The value of enhanced MR radiomics in estimating the IDH1 genotype in high-grade gliomas. BioMed Res Int. (2020) 2020:1–6. doi: 10.1155/2020/4630218 PMC7604586 33163535

[21] Cao M Suo S Zhang X Wang X Xu J Yang W . Qualitative and quantitative MRI analysis in IDH1 genotype prediction of lower-grade gliomas: A machine learning approach. BioMed Res Int. (2021) 2021:1235314. doi: 10.1155/2021/1235314 33553421 PMC7847347

[22] Huang WY Wen LH Wu G Hu MZ Zhang CC Chen F . Comparison of radiomics analyses based on different magnetic resonance imaging sequences in grading and molecular genomic typing of glioma. J Comput Assist Tomogr. (2021) 45:110–20. doi: 10.1097/RCT.0000000000001114 33475317

[23] Manikis GC Ioannidis GS Siakallis L Nikiforaki K Iv M Vozlic D . Multicenter DSC-MRI-based radiomics predict IDH mutation in gliomas. Cancers (Basel). (2021) 13:3965. doi: 10.3390/cancers13163965 34439118 PMC8391559

[24] Hrapşa I Florian IA Şuşman S Farcaş M Beni L Florian IS . External validation of a convolutional neural network for IDH mutation prediction. Medicina (Kaunas). (2022) 58:526. doi: 10.3390/medicina58040526 35454365 PMC9025144

[25] Kandalgaonkar P Sahu A Saju AC Joshi A Mahajan A Thakur M . Predicting IDH subtype of grade 4 astrocytoma and glioblastoma from tumor radiomic patterns extracted from multiparametric magnetic resonance images using a machine learning approach. Front Oncol. (2022) 12:879376. doi: 10.3389/fonc.2022.879376 36276136 PMC9585657

[26] Wang H Zhang S Xing X Yue Q Feng W Chen S . Radiomic study on preoperative multi-modal magnetic resonance images identifies IDH-mutant TERT promoter-mutant gliomas. Cancer Med. (2023) 12:2524–37. doi: 10.1002/cam4.5097 PMC9939206 36176070

[27] Wang J Hu Y Zhou X Bao S Chen Y Ge M . A radiomics model based on DCE-MRI and DWI may improve the prediction of estimating IDH1 mutation and angiogenesis in gliomas. Eur J Radiol. (2022) 147:110141. doi: 10.1016/j.ejrad.2021.110141 34995947

[28] Sudre CH Panovska-Griffiths J Sanverdi E Brandner S Katsaros VK Stranjalis G . Machine learning assisted DSC-MRI radiomics as a tool for glioma classification by grade and mutation status. BMC Med Inf Decision Making. (2020) 20. doi: 10.1186/s12911-020-01163-5 PMC7336404 32631306

[29] Cuocolo R Cipullo MB Stanzione A Romeo V Green R Cantoni V . Machine learning for the identification of clinically significant prostate cancer on MRI: a meta-analysis. Eur Radiol. (2020) 30(12):6877–87. doi: 10.1007/s00330-020-07027-w 32607629

[30] van Kempen EJ Post M Mannil M Witkam RL Laan MT Patel A . Performance of machine learning algorithms for glioma segmentation of brain MRI: a systematic literature review and meta-analysis. Eur Radiol. (2021) 31(12):9631–58. doi: 10.1007/s00330-021-08035-0 PMC8589805 34019128

[31] Zhao J Huang Y Song Y Xie D Hu M Qiu H . Diagnostic accuracy and potential covariates for machine learning to identify IDH mutations in glioma patients: evidence from a meta-analysis. Eur Radiol. (2020) 30(8):4664–74. doi: 10.1007/s00330-020-06717-9 32193643

[32] Shibahara I Sonoda Y Shoji T Kanamori M Saito R Inoue T . Malignant clinical features of anaplastic gliomas without IDH mutation. Neuro Oncol (2015) 17(1):136–44. doi: 10.1093/neuonc/nou112 PMC4483044 24958096

[33] Halligan S Menu Y Mallett S . Why did European adiology reject my radiomic biomarker paper? How to correctly evaluate imaging biomarkers in a clinical setting. Eur Radiol. (2021) 31:9361–8. doi: 10.1007/s00330-021-07971-1 PMC8589811 34003349

[34] Kim DW Jang HY Kim KW Shin Y Park SH . Design characteristics of studies reporting the performance of artificial intelligence algorithms for diagnostic analysis of medical images: results from recently published papers. Korean J Radiol. (2019) 20:405–10. doi: 10.3348/kjr.2019.0025 PMC6389801 30799571

