# Supplementary material for: Diagnostic accuracy of a machine learning-based radiomics approach of MR in predicting IDH mutations in glioma patients: a systematic review and meta-analysis
Source: Front Oncol. 2024 Jul 30;14:1409760. doi: 10.3389/fonc.2024.1409760 (PMC11319127; doi:10.3389/fonc.2024.1409760)
Supplement: Supplementary file 1 [file Table_1.docx]

| **No.** | **Search Query for PubMed** | **Result** |
| --- | --- | --- |
| #1 | "Glioma"[MeSH Terms] OR "Oligodendroglioma"[MeSH Terms] OR "Astrocytoma"[MeSH Terms] | 97155 |
| #2 | "Gliomas"[Title/Abstract] OR "Oligodendrogliomas"[Title/Abstract] OR "Astrocytomas"[Title/Abstract] OR "astroglioma*"[Title/Abstract] OR "astrocytic glioma*"[Title/Abstract] | 35985 |
| #3 | **#1 OR #2** | 105760 |
| #4 | "Isocitrate Dehydrogenase"[MeSH Terms] | 7569 |
| #5 | "isocitrate dehydrogenase i"[Title/Abstract] OR "isocitrate dehydrogenase i"[Title/Abstract] OR "IDH"[Title/Abstract] | 5193 |
| #6 | **#4 OR #5** | 10714 |
| #7 | "Artificial Intelligence"[MeSH Terms] OR "Machine Learning"[MeSH Terms] OR "Deep Learning"[MeSH Terms] | 165554 |
| #8 | "computational intelligence"[Title/Abstract] OR "machine intelligence"[Title/Abstract] OR "computer reasoning"[Title/Abstract] OR "AI"[Title/Abstract] OR "computer vision system*"[Title/Abstract] OR "knowledge acquisition"[Title/Abstract] OR "knowledge representation*"[Title/Abstract] OR "transfer learning"[Title/Abstract] OR "machine learning"[Title/Abstract] OR "hierarchical learning"[Title/Abstract] OR "deep learning"[Title/Abstract] | 159811 |
| #9 | **#7 OR #8** | 267521 |
| #10 | "radiomics"[Title/Abstract] OR "radiogenomic*"[Title/Abstract] | 7374 |
| #11 | **#9 OR #10** |  |
| #12 | \| "Magnetic Resonance Imaging"[MeSH Terms] \|  \| \| --- \| --- \| | 525060 |
| #13 | "MRI"[Title/Abstract] OR "mri scans"[Title/Abstract] | 310699 |
| #14 | **#12 OR #13** | 272012 |
| #15 | **#3 AND #6 AND # 11 AND #14** | 160 |

| **No.** | **Search Query for Embase** | **Result** |
| --- | --- | --- |
| #1 | 'glioma'/exp OR 'oligodendroglioma'/exp OR 'astrocytoma'/exp | 170281 |
| #2 | gliomas:ab,ti OR oligodendrogliomas:ab,ti OR astrocytomas:ab,ti OR astroglioma*:ab,ti OR 'astrocytic glioma*':ab,ti | 51079 |
| #3 | **#1 OR #2** | 175353 |
| #4 | 'isocitrate dehydrogenase'/exp | 8435 |
| #5 | 'isocitrate dehydrogenase i':ab,ti OR 'isocitrate dehydrogenase-i':ab,ti OR idh:ab,ti | 8716 |
| #6 | **#4 OR #5** | 15190 |
| #7 | 'deep learning'/exp OR 'machine learning'/exp OR 'artificial intelligence'/exp | 389480 |
| #8 | **'**computational intelligence'/exp OR 'computational intelligence' OR 'machine intelligence'/exp OR 'machine intelligence' OR 'computer reasoning'/exp OR 'computer reasoning' OR ai OR 'computer vision system*' OR 'knowledge acquisition'/exp OR 'knowledge acquisition' OR 'knowledge representation*' OR 'transfer learning, machine learning' OR 'hierarchical learning'/exp OR 'hierarchical learning' OR 'deep learning':ab,ti | 866380 |
| #9 | **#7 OR #8** | 1133394 |
| #10 | **'**radiomics'/exp OR radiomics OR radiogenomic*:ab,ti | 10911 |
| #11 | **#9 OR #10** | 1138055 |
| #12 | 'magnetic resonance imaging'/exp OR 'magnetic resonance imaging' OR 'mri'/exp OR mri OR 'mri scans':ab,ti | 1266649 |
| #13 | **#3 AND #6 AND #11 AND #12** | 279 |

| **No.** | **Search Query for Web of Science** | **Result** |
| --- | --- | --- |
| #1 | TS=("Glioma" OR "Oligodendroglioma" OR "Astrocytoma" OR "Gliomas" OR "Oligodendrogliomas" OR "Astrocytomas" OR "Astroglioma*" OR "Astrocytic Glioma*") | 117064 |
| #2 | TS=("Isocitrate Dehydrogenase" OR "Isocitrate Dehydrogenase I" OR "Isocitrate Dehydrogenase-I" OR "IDH") | 11743 |
| #3 | TS=("Artificial Intelligence" OR "Machine Learnin" OR "Deep Learning" OR "Computational Intelligence" OR "Machine Intelligence" OR "Computer Reasoning" OR "AI" OR "Computer Vision System*" OR "Knowledge Acquisition" OR "Knowledge Representation*" OR "Transfer Learning" OR "machine learning" OR" Hierarchical Learning" OR "deep learning") | 613498 |
| #4 | TS=("Magnetic Resonance Imaging" OR MRI OR "MRI Scans" OR radiomics OR radiogenomic*) | 8111625 |
| #5 | #1 AND #2 AND #3 AND #4 | 198 |

| **No.** | **Search Query for Cochrane Library** | **Result** |
| --- | --- | --- |
| #1 | MeSH descriptor: [Glioma] explode all trees OR MeSH descriptor: [Oligodendroglioma] explode all trees OR MeSH descriptor: [Astrocytoma] explode all trees | 1685 |
| #2 | (Gliomas):ti,ab,kw OR (Oligodendrogliomas):ti,ab,kw OR (Astrocytomas):ti,ab,kw OR (Astroglioma*):ti,ab,kw OR (Astrocytic Glioma*):ti,ab,kw (Word variations have been searched) | 2324 |
| #3 | **#1 OR #2** | 3052 |
| #4 | MeSH descriptor: [Isocitrate Dehydrogenase] explode all trees | 67 |
| #5 | (Isocitrate Dehydrogenase I):ti,ab,kw OR (Isocitrate Dehydrogenase-I):ti,ab,kw OR (IDH):ti,ab,kw (Word variations have been searched) | 349 |
| #6 | **#4 OR #5** | 388 |
| #7 | MeSH descriptor: [Machine Learning] explode all trees OR MeSH descriptor: [Deep Learning] explode all trees OR MeSH descriptor: [Artificial Intelligence] explode all trees | 2391 |
| #8 | (Knowledge Acquisition):ti,ab,kw OR (Knowledge Representation*):ti,ab,kw OR (Transfer Learning):ti,ab,kw OR (machine learning):ti,ab,kw OR (Hierarchical Learning):ti,ab,kw (Word variations have been searched) OR (Computational Intelligence):ti,ab,kw OR (Machine Intelligence):ti,ab,kw OR (Computer Reasoning):ti,ab,kw OR (AI):ti,ab,kw OR (Computer Vision System*):ti,ab,kw (Word variations have been searched) OR (deep learning):ti,ab,kw (Word variations have been searched) | 16953 |
| #9 | **#7 OR #8** | 18283 |
| #10 | (radiogenomic*):ti,ab,kw OR (radiomics):ti,ab,kw (Word variations have been searched) | 531 |
| #11 | **#9 OR #10** | 18645 |
| #12 | MeSH descriptor: [Magnetic Resonance Imaging] explode all trees | 10609 |
| #13 | (MRI):ti,ab,kw OR (MRI Scans):ti,ab,kw (Word variations have been searched) | 30202 |
| #14 | **#12 OR #13** | 35080 |
| #14 | #3 AND #6 AND #11 AND #14 | 4 |
