# Supplementary material for: Diagnostic accuracy of a machine learning-based radiomics approach of MR in predicting IDH mutations in glioma patients: a systematic review and meta-analysis
Source: Front Oncol. 2024 Jul 30;14:1409760. doi: 10.3389/fonc.2024.1409760 (PMC11319127; doi:10.3389/fonc.2024.1409760)
Supplement: Supplementary file 2 [file Table_2.docx]

Table 4

The results of subgroup analyses.

| Analysis | No.of studies | Sensitivity | Specificity | PLR | NLR | Diagnostic odds ratio | AUC |
| --- | --- | --- | --- | --- | --- | --- | --- |
| Modeling methods | | | | | | |  |
| DL | 2 | 0.91(0.76.0.98) | 0.77 (0.55, 0.92) | 3.72 (1.68, 8.26) | 0.14(0.02, 0.82) | 27.85(3.23,240.01) | 0.50 |
| ML | 12 | 0.73(0.68,0.77) | 0.83(0.79,0.86) | 3.65(2.64.5.049) | 0.34(0.25,0.46) | 17.98(8.99,35.99) | 0.88 |
| Clinical information | | | | | | |  |
| with | 3 | 0.89（0.72，0.98） | 0.79（0.66，0.88） | 3.80（2.32，6.15） | 0.19(0.07,0.49) | 27.65(6.82,112.08) | 0.91 |
| without | 11 | 0.73（0.68，0.78） | 0.83（0.79,0.86） | 3.59（2.52，5.11） | 0.33(0.24,0.47) | 17.57(8.55,36.10) | 0.88 |
| Glioma grade | | | | | | |  |
| LGG | 3 | 0.93(0.85.0.98) | 0.71(0.48,0.89) | 2.88(1.53.5.40) | 0.10(0.02,0.45) | 30.73(6.77, 139.56) | 0.81 |
| HGG | 3 | 0.71(0.58.0.83) | 0.91(0.85,0.95) | 8.61(1.70,43.51) | 0.37(0.21,0.67) | 30.95 (11.01, 86.99) | 0.91 |
| Validation | | | | | | |  |
| External validation | 5 | 0.78(0.70,0.85) | 0.83(0.72,0.91) | 3.20(1.89,5.43) | 0.231 (0.08, 0.61) | 20.32(5.05,81.73) | 0.89 |
| Internal validation | 6 | 0.81(0.74,0.87) | 0.87(0.82,0.91) | 4.51(2.79,7.32) | 0.26(0.16,0.43) | 26.32(13.00,53.26) | 0.89 |

PLR, positive likelihood ratio; NLR, negative likelihood ratio; DOR, diagnostic odds ratio; SVM,support vector machines; LR,logistic regressi
